# Current Programs and Incentives to Overcome Rural Physician Shortages in the United States: A Narrative Review

**DOI:** 10.1007/s11606-023-08122-6

**Published:** 2023-06-20

**Authors:** Kelley Arredondo, Hilary N. Touchett, Sundas Khan, Matthew Vincenti, Bradley V. Watts

**Affiliations:** 1 Houston VA HSR&D Center for Innovations in Quality, Effectiveness, and Safety, Michael E. DeBakey VA Medical Center, 2450 Holcombe Blvd Suite 01Y, Houston, TX 77021 USA; 2 Department of Medicine, Baylor College of Medicine, Houston, TX USA; 3 VHA Office of Rural Health’s Veterans Resource Center, White River Junction, VT USA; 4 South Central Mental Illness Research, Education, Clinical Center, a Virtual Center, Little Rock, AR USA; 5 Department of Medicine, Dartmouth Geisel School of Medicine, Hanover, NH USA; 6 Department of Psychiatry, Dartmouth Geisel School of Medicine, Hanover, NH USA

**Keywords:** physician shortages, rural health, rural incentives, rural workforce, rural programs

## Abstract

Access to healthcare continues to be a top priority and prominent challenge in rural communities, with 20% of the total U.S. population living in rural areas while only 10% of physicians practice in rural areas. In response to physician shortages, a variety of programs and incentives have been implemented to recruit and retain physicians in rural areas; however, less is known about the types and structures of incentives that are offered in rural areas and how that compares to physician shortages. The purpose of our study is to conduct a narrative review of the literature to identify and compare current incentives that are offered by rural physician shortage areas to better understand how resources are being allocated to vulnerable areas. We reviewed published peer-reviewed articles from 2015–2022 to identify incentives and programs designed to address physician shortages in rural areas. We augment that review by examining the gray literature, including reports and white papers on the topic. Identified incentive programs were aggregated for comparison and translated into a map that depicts high, medium, and low levels of geographically designated Health Professional Shortage Areas (HPSAs) and the number of incentives offered by state. Surveying current literature regarding different types of incentivization strategies while comparing to primary care HPSAs provides general insights on the potential influence of incentive programs on shortages, allows easy visual review, and may provide greater awareness of available support for potential recruits. Providing a broad overview of the incentives offered in rural areas will help illuminate whether diverse and appealing incentives are offered in the most vulnerable areas and guide future efforts to address these issues.

**Supplementary Information:**

The online version contains supplementary material available at 10.1007/s11606-023-08122-6.

## Introduction

Despite the fact that 20% of Americans live in rural communities, only 10% of physicians work in rural areas, and a mere 1% of graduate medical training programs in the United States are located in rural communities.^1^ In 2019, there were 1.6 times more primary care physicians per 10,000 people in urban U.S. counties, than rural counties.^2^ Further exacerbating this maldistribution, a third of physicians are expected to retire in the next decade^3^, with half of the rural physician workforce already over the age of 55.^4^ Therefore, public and private entities have invested considerable resources towards addressing rural physician shortages. These rural physician shortages have persisted for decades, negatively impacting the health outcomes of over 50 million Americans residing in rural areas.^5,6^

Many rural communities are designated Health Professional Shortage Areas (HPSAs); the intersection of rural and health professional shortage area negatively impacts timely access to care, access to specialty care and ancillary health services, and overall health status.^7,8^ Rural residents are older and sicker than their urban counterparts, travel longer and further for their care, and have higher rates of morbidity and mortality.^9^ Since access to primary healthcare plays a crucial role in overall health and wellbeing, physician shortages have broad impacts on rural residents’ health, life expectancy, and quality of life.

To promote healthcare access in HPSAs, a myriad of approaches across organizations and agencies have been implemented. For example, some have posed solutions like virtual care options such as video telehealth to improve access to specialty care^10^; however, this solution only partially meets this need.^11^ Many rural residents prefer in-person modalities and/or lack sufficient access to broadband for high-quality video conference, and many healthcare needs require in-person assessment and treatment, making recruitment and retention of providers in these areas important elements to addressing rural health disparities.^12^

Many programs and funding mechanisms have been introduced to support rural health workforce development over the past 3 decades. The structure and components of these programs and incentives vary. Less is known about whether diverse and appealing incentives are offered in the most vulnerable areas and how these incentives compare to one another. The variability across programs makes it difficult to discern appealing and effective components; therefore, our study addresses the following research questions:

- What are the most common types of incentives and programs offered to recruit and/or retain physicians in HPSAs?
- Do programs and incentives give preference to known predictors (e.g., rural upbringing) of rural physician recruitment and retainment?
- Are programs and incentives offered in states with the greatest rural physician shortages?

We surveyed publicly available rural health workforce resources and published rural workforce incentivization literature and compared these to federally designated geographic primary care HPSAs. We compared programs and incentives across states that recruit and retain physicians, rather than nurse practitioners and/or physician assistants, because physician training, licensing, and practice authority standards are consistent across states. Nurse practitioners and physician assistants have much greater variability in their scope and practice authority across states due to policy variations that likely impact program availability and efficacy of recruitment and retainment efforts.^13,14^ This issue warrants a more nuanced examination than this overview provides and needs more research. However, we do include programs and incentives open to physicians and additional healthcare providers and excluded programs and incentives that did not include physicians. Our article extends our understanding of incentives and programs offered to recruit and retain physicians in rural areas and provides an overview of the differences in incentives and stipulations that may influence physicians’ decision to pursue an appointment in a rural area. This information can help inform program leadership and policymakers interested in developing or funding rural physician recruitment and retainment efforts.

## Methods

We conducted a narrative review of the published and grey literature on interventions and programs utilized to recruit and retain physicians in HPSAs. A narrative review allows more latitude in addressing our multiple research questions using varied data sources.^15,16^

### Search Strategy

Incentives and programs were identified through the Rural Health Information (RHI) Hub accessed in July and August 2022. The RHI Hub offers a repository of funding and opportunities available through different sponsors in various states and nationwide; however, the website contains only programs and incentives that were recently offered or are currently recruiting, which allowed us to identify only programs offered between January 2020 and July 2022. Once on the website, we searched Rural Funding and Opportunities by type, which included: Awards (Monetary), Educational Opportunities and Fellowships, Incentives, Loan Repayment Programs (LRPs), Loans, and Scholarships. Individual program websites were then used to gather program specific information.

For the published literature, PubMed and Google Scholar were searched in August 2022 using the following terms: *physician shortage, rural health, rural incentives, rural workforce,* and *rural physician program*. Additionally, each unique program identified during the grey literature search was searched to capture published outcomes on identified programs.

### Program, Incentive, and Study Selection Criteria

Programs/incentives included in this review were a) offered in the United States; b) open to physicians; c) focused on recruiting or retaining physicians in rural areas; and for the gray literature d) actively accepting applicants sometime between January 2020 and July 2022 (due to web-based information availability), or for the published literature, e) published between 2015 and 2022.

### Data Extraction

#### Programs and Incentives

Data were extracted from each program, incentive, and published report. Data were aggregated using spreadsheets and reviewed by 2 authors for consensus (KA and SK); discrepancies were resolved by a third author (HT). Data extracted from each incentive/program included: a) funding/incentive type; b) funding/incentive mechanism; c) recruitment vs. retention focus; d) geographic area; e) service time commitment; f) whether the incentive/program prefers physicians that are from rural areas; and g) preferred physician specialty.

#### HPSAs

Data from the Health Resources and Services Administration were utilized to identify all geographic primary care HPSAs in rural and partially rural areas. Primary care HPSA scores are evaluated on population to provider ratio, percent of individuals below the federal poverty level, infant health index, and average travel time to nearest source of care. Scores range from 1 to 25, with higher scores indicating greater priority. We classified HPSAs with a rating from 1–13 as low priority, 14–17 as moderate priority, and 18 + as high priority. We compared known HPSAs to available programs and incentives by generating frequency counts for HPSAs with a rating of 18 + and categorized states as having 0, 1–5, 6–12, or 13 or more HPSAs 18 + .

## Results

A total of 247 programs and incentives were included and coded by funding/incentive type: educational opportunities and fellowships (n = 89), LRPs (n = 70), J-1 visa waivers (n = 48), scholarships (n = 26), and financial incentives (i.e., any financial incentive that was not a scholarship or LRP; n = 14). All data generated or analyzed during this study are included in the supplementary data file.

### Description of Programs and Incentives

Overall, 120 programs/incentives were available exclusively to physicians, while a little over half (n = 127) were available to physicians and other healthcare professionals. Most programs and incentives were focused on recruiting providers to rural areas (n = 218). Fewer programs and incentives offered resources or incentives to providers who were already practicing in rural areas (i.e., retainment; n = 24), and only 5 programs and incentives focused on both recruitment and retainment. Table 1 shows the frequency count of programs/incentives offered at each stage of the student to rural physician pipeline. Of the programs and incentives that specified a preference (n = 130), 125 preferred physicians with a primary care focus, which generally included family medicine, internal medicine, pediatrics, and geriatrics. Other preferred specialties included obstetrics and gynecology (n = 57), psychiatry (n = 63), and general surgery (n = 11) as these specialties often serve essential healthcare roles in primary care. Using census geographic areas, most programs were offered in the South (n = 88) and the least were in the Northeast (n = 33; see Table 2). Program count offered by state and HPSA 18 + designation can be seen in the Figure.

**Table 1:** Frequency Count of What Stage in the Student to Physician Pipeline Programs and Incentives Target

Stage	Frequency
High School or Earlier	11
Undergraduate	18
Medical School	85
Residency	63
Early Career	3
Leadership	6
Licensed Physician*	69
Non-specified stage of career	8

*Note. *Some programs and incentives were open to individuals at multiple stages, therefore the total count exceeds the total number of individual programs and incentives. *Just specifies that applicants must have a practicing license

**Table 2:** Program and Incentive Frequency Count By Geographic Region and Number of HPSAs in Each Region By HPSA Score

Region	States	Programs	HPSAs 1–13	HPSAs 14–17	HPSAs 18 +
Northeast	Connecticut; Maine; Massachusetts; New Hampshire; New Jersey; New York; Pennsylvania; Rhode Island; Vermont	33	34	7	0
Midwest	Illinois; Indiana; Iowa; Kansas; Michigan; Minnesota; Missouri; Nebraska; North Dakota; Ohio; South Dakota; Wisconsin	68	270	66	11
South	Alabama; Arkansas; Delaware; District of Columbia; Florida; Georgia; Kentucky; Louisiana; Maryland; Mississippi; North Carolina; Oklahoma; South Carolina; Tennessee; Virginia; West Virginia; Texas	88	276	197	78
West	Alaska; Arizona; California; Colorado; Hawaii; Idaho; Montana; Nevada; New Mexico; Oregon; Utah; Washington; Wyoming	74	165	140	24

*Note. *Some programs and incentives were offered in multiple geographic regions; therefore, the total count exceeds the total number of individual programs and incentives

**Figure 1:**
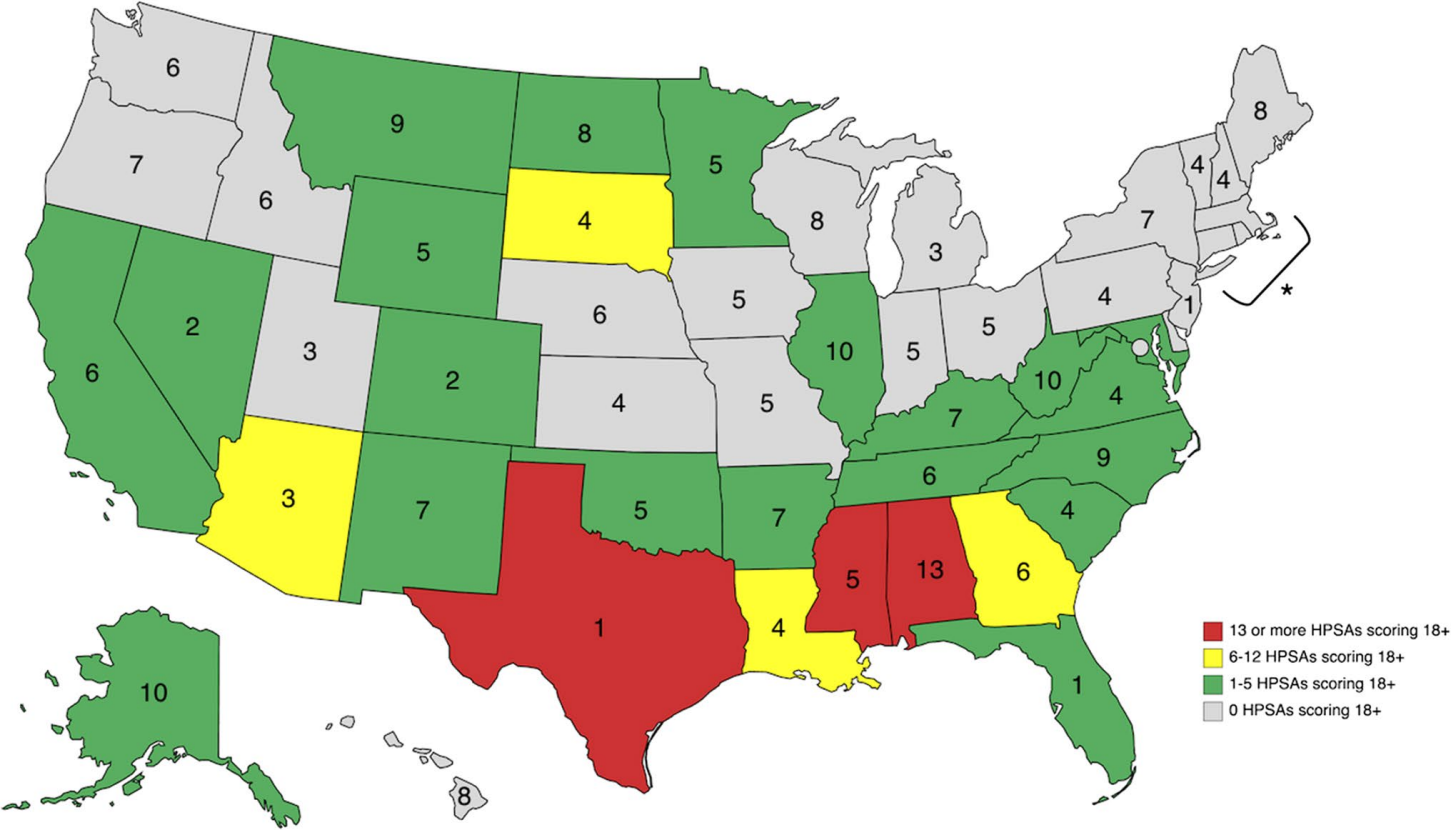
Program and incentive frequency count with geographic primary care Health Professional Shortage Areas (HPSAs)

Programs and incentives were further coded into funding/incentive type; for example, “educational opportunities and fellowships” included scholar programs, educational experiences, and leadership training. Overall, the largest portion of incentives were LRPs (28.3%), followed by J-1 visa waivers (19.4%), scholar programs (11.3%), scholarships (10.5%), and educational programs (10.5%). The remaining funding and incentive types included but were not limited to, educational experiences (3.6%), fellowships (3.2%), and leadership training (2.8%). We discuss the 3 most common program and incentive types (i.e., LRPs, J-1 Visa Waivers, and scholar programs) below Fig. 1.

### Loan Repayment Programs

Loan repayment programs were the most common type of incentive program offered (n = 70). Although a variety of LRPs exist, the most common are State LRPs. Loan repayment programs aim to recruit physicians by offering loan repayment in exchange for service in a shortage area. These programs do not have application fees and may offer loans that will be waived once a service commitment is fulfilled or provide practicing physicians with loan repayment for qualifying loans as each year of service commitment is completed. Thus, LRPs are typically offered in exchange for an equal amount of service commitment; for example, 2 years of loan repayment assistance may require 2 years of service in a shortage area. The time commitment for the LRPs identified in the literature ranged from 1 to 9 years. Most programs (n = 52, 74%) did not specify a preference for any type of applicants’ background. However, some LRPs did specify a preference or requirement such as applicants attending a university or college in the state or area (n = 9) or applicants that were state residents or from a specific region (e.g., Appalachia; n = 7). Almost no LRPs stated a preference for applicants from underrepresented or underserved groups (n = 2) or rural areas (n = 1). Most LRPs were offered to fully licensed physicians (n = 53), although some were open to medical students (n = 8), or residents (n = 5). Notably, some programs and incentives were open to undergraduate students (n = 2). Loan repayment programs ranged from $20,000 to $250,000 in loan forgiveness, typically corresponding to length of service.

### J-1 Visa Waiver Programs

In an effort to attract international medical graduates to HPSAs, J-1 visa waivers are frequently offered by states; these waivers eliminate the requirement of J-1 visa holders who must return to their home countries for at least 2 years once they complete their training.^17,18^ Instead, the J-1 visa waivers allows international medical students to practice in the U.S. immediately after completing their training. Each state participating in the Conrad J-1 Visa Waiver Program is allocated 30 slots, and some states are allocated additional flex slots that can be used to fulfill additional needs, such as mental health professions shortage areas, which may bring the total slots available to 40.^18^ Part of the stipulation of the waiver program is that each potential facility that applies to sponsor an international medical graduate must demonstrate that sufficient efforts were made to recruit physicians that are U.S. citizens or residents. Across all identified J-1 Visa waiver programs (n = 48), each international medical graduate must commit to 3 years of service in a HPSA and can apply for the program during their residency training by acquiring sponsorship from an eligible facility. The program does not offer any additional financial incentives and although most programs are free for physicians to apply to (n = 38), some states do require a non-refundable application fee (n = 10) that ranged from $200 to $3,571.

### Scholar Programs

The 28 scholar programs identified were Area Health Education Centers (AHECs) that are offered through local, statewide, and regional efforts in conjunction with the National AHEC Organization.^19^ The purpose of the AHEC Scholars program is to recruit physicians who have an interest in rural and/or underserved areas by offering education, training, and exposure to rural and underserved areas.^12^ Physician applicants must be in medical school, 2 years from graduating, and able to commit to the 2-year program, which consists of 40 hours of experiential and 40 hours of didactic activities, in addition to medical school coursework. Twenty-seven programs were available only to applicants attending a specific university/college (e.g., applicants must be attending University of Arkansas) or at a minimum, a university/college within the state; and 3 programs preferred applicants from underrepresented, disadvantaged groups, or those from rural areas. Of the programs that offered stipends (n = 13), some stated a non-specified stipend amount (n = 4) or simply stated there was the possibility of stipend support (n = 3). These programs compete with programs that explicitly state financial support; for example, 1 program offered $1,000 for the first 120 students to enroll in the program, and 5 programs offered specific stipend amounts ranging from $500 to $750 per year. Lastly, almost all scholar programs were focused on recruiting and 1 program offered continuing education opportunities for established practicing physicians in an effort to retain rural physicians.

## Discussion

We conducted a narrative review to better understand the types of programs and incentives currently offered to recruit and retain physicians in rural areas. Although many of the programs and incentives identified in the narrative review have been around for decades, little is known about how many programs are offered in each state compared to shortage areas and how programs compare to one another, regarding monetary awards, service commitment required, and the kinds of incentives and programs offered at different stages of the student to physician pipeline. Below we discuss implications by the main objectives of the study.

### What are the Most Common Types of Incentives and Programs Offered to Recruit and/or Retain Physicians to Shortage Areas?

The most prevalent incentive types were LRPs, followed by J-1 visa waivers, scholar programs, education programs, and scholarships. While these incentives initially seem appealing for physicians, barriers may exist that make these incentives difficult to obtain. For example, most scholarship programs are available to medical students and require a service commitment in a HPSA in exchange for the scholarship; however, the amount that scholarships offer tends to be lower than the amount offered through LRPs. This is problematic because many programs do not allow applicants to apply if they have service obligations to another award. That is, physicians who are fulfilling service commitments from scholarships they received during medical school may not qualify for LRPs until after they have fulfilled their initial service obligation. Given that rural physicians earn less than their urban counterparts but have the same amount of debt^20^, programs should offer more complementary support so that students and physicians can take advantage of these different programs. These programs compete with similar benefits offered by the private sector; in fact, a systematic review on the attrition of physicians in the public sector identified financial incentives as one of the main drivers of physicians’ choice between the 2 sectors.^21^ Collectively, these elements contribute to physicians’ decision making when choosing which program or incentive is the best fit for them or whether practicing in an urban setting is best.

Interestingly, we found that many programs and incentives may state that their objective is to help with both recruitment and retainment, but few are available to physicians already practicing in rural areas. For example, most programs and incentives had a focus on recruitment (n = 218) with only 24 focused on retainment and 5 offering both recruitment and retainment resources. The literature supports that many physicians leave HPSAs once their service commitment contract is up, taking with them institutions’ investment and negatively impacting patients’ continuity of care;^22,23^ thus, more programs and incentives should focus on retainment. This review illuminates the scarcity of programs and incentives offered to physicians already practicing in rural areas.

### Do Programs and Incentives Give Preference to Known Predictors of Rural Physician Recruitment and Retainment?

Despite the literature supporting that students with rural backgrounds have a higher probability of staying in rural areas to practice, only 20 programs and incentives explicitly stated preferring applicants from rural area. In fact, most programs and incentives did not specify preferring any type of applicant, whether they be from an underrepresented group or in state resident. Giving preference to applicants from rural areas or applicants from within the state helps increase the probability that physicians will remain in the rural area, or at a minimum, within the state. Beyond recruitment of students from rural areas, identifying with rural culture also increases the probability of choosing rural practice.^21,24,25^ This can be fostered through rural health experiences, such as training students in the rural areas where they need students to practice.^21^

Most programs were offered at the medical school stage or later, while only 7 programs were offered to high school students or younger. One way to foster students’ identification with rural culture is through the early introduction of education and incentive programs in rural settings. Presenting non-rural students with opportunities to engage in rural recreational activities and life can foster a rural identity, increasing the likelihood of these individuals practicing in rural areas.^21^ Additionally, providing individuals with realistic job previews during the recruitment and hiring process has a significant positive relationship with job satisfaction and decreases turnover.^26^ Rural residency rotations may act as a realistic job preview, which allows residents to establish expectations about the environment, healthcare facility, and demands.^27^ Our study identified only 4 programs/incentives that gave preference to applicants who did their residency in state. Offering rural residency rotations in rural areas and giving preference to these applicants to incentives and programs once they enter the workforce may increase the probability of physicians remaining in rural areas even after their service commitment is met. Thus, organizations and institutions should focus their efforts on developing programs and incentives to introduce rural healthcare to younger students as well as incorporating rural residency rotations to enhance both recruitment and retention.

### Are Programs and Incentives Offered in States With the Most Shortage Areas?

By region, the South and West have the highest number of high-priority HPSAs with scores 18 and higher (n = 78 and n = 24, respectively) and the highest number of programs/incentives available (n = 88 and n = 74, respectively). However, discrepancies within regions arise when we examine program/incentive allocation at the state level. While there are several nationwide programs available to applicants in the U.S., many programs are limited to specific states, and those do not always reflect areas of highest need. For example, when looking at number of HPSAs scoring 18 or higher within each state, Mississippi (n = 16), Texas (n = 14), and Alabama (n = 13) have the greatest need for programs that recruit/retain physicians. Yet, Mississippi has 5 programs, Texas has only 1 program, while Alabama has more programs than any other state (n = 13). This maldistribution of programs can further exacerbate shortages by lessening the appeal of working in a HPSA when similar benefits are available elsewhere. Additionally, considering that rural scholar programs are a common recruitment strategy used to foster community ties in rural areas and that approximately 57% of physicians remain in the state where they complete training^28^, programs (and their distribution) play a vital role in filling HPSA needs.

### Limitations and Future Direction

This study has limitations. It relies on publicly available data that was accessed in July through August of 2022. Programs that did not have a publicly accessible recruitment information during our search were not identified in the literature review. Thus, our review provides a snapshot of programs and incentives currently offered, and future research should investigate trends of previous programs and incentives offered in comparison to current programs and incentives to see how efforts to recruit and retain providers have changed over time. Additionally, merging the information of programs offered by state and federally designated HPSAs presented some challenges due to variability in how geographic areas are defined across data sources (e.g., many sources use county level data which is not always equivalent to regions granted a geographic HPSA designation). Lastly, there was limited program evaluation data in the published or gray literature that would have aided in determining whether these efforts have increased physician recruitment and retention. Future efforts should investigate the effectiveness of these interventions and determine what common aspects of these programs and interventions differentiate them from those that are less successful.

## Conclusion

Although efforts have been implemented across states for many years, rural physician shortages persist and are projected to get worse.^3,4^ We sought to coalesce information across different programs and incentives to get a better understanding of how these programs compare with one another. While this is an important first step, little is known about the success rates of specific programs and interventions in retaining physicians in rural areas. In order to truly understand which programs and interventions are successful in their mission to increase physicians in HPSAs, standardized program evaluations and retention rate reporting across years are necessary.

## Supplementary Information:

Below is the link to the electronic supplementary material.

Supplementary file1 (XLSX 145 kb)
